# *trans*-Dichloridobis(4-nitro­aniline-κ*N* ^1^)palladium(II)

**DOI:** 10.1107/S1600536812042134

**Published:** 2012-10-13

**Authors:** Tian-Jun Feng

**Affiliations:** a College of Mathematics, Physics and Software Engineering, Lanzhou Jiaotong University, Lanzhou 730070, People’s Republic of China

## Abstract

In the title compound, [PdCl_2_(C_6_H_6_N_2_O_2_)_2_], the Pd^II^ atom is coordinated in a distorted square-planar geometry by two N atoms from two 4-nitro­aniline ligands and two Cl atoms in a *trans* arrangement. Inter­molecular N—H⋯Cl hydrogen bonds involving the amino groups and chloride anions lead to a chain along [100]. These chains are further self-assembled into a three-dimensional network through N—H⋯O and N—H⋯Cl hydrogen bonds.

## Related literature

For background to the application of palladium compounds in catalysis, see: Hartley (1973 ▶); Padmanabhan *et al.* (1985 ▶). For related structures, see: Chen *et al.* (2002 ▶); Newkome *et al.* (1982 ▶).
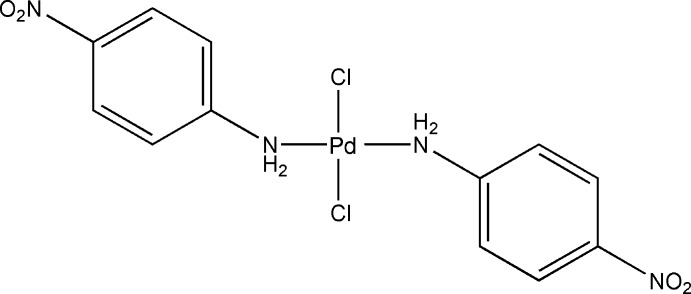


## Experimental

### 

#### Crystal data

[PdCl_2_(C_6_H_6_N_2_O_2_)_2_]
*M* *_r_* = 453.56
Monoclinic, 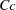

*a* = 5.6014 (8) Å
*b* = 26.246 (4) Å
*c* = 11.0763 (16) Å
β = 99.828 (2)°
*V* = 1604.5 (4) Å^3^
*Z* = 4
Mo *K*α radiation
μ = 1.51 mm^−1^
*T* = 296 K
0.33 × 0.30 × 0.26 mm

#### Data collection

Bruker APEXII CCD diffractometer
Absorption correction: multi-scan (*SADABS*; Sheldrick, 1996 ▶) *T*_min_ = 0.626, *T*_max_ = 0.688
4453 measured reflections
2135 independent reflections
2075 reflections with *I* > 2σ(*I*)
*R*_int_ = 0.018

#### Refinement

*R*[*F* ^2^ > 2σ(*F* ^2^)] = 0.021
*wR*(*F* ^2^) = 0.077
*S* = 1.06
2135 reflections
208 parameters
2 restraints
H-atom parameters constrained
Δρ_max_ = 0.50 e Å^−3^
Δρ_min_ = −0.37 e Å^−3^
Absolute structure: Flack (1983 ▶), 696 Friedel pairs
Flack parameter: 0.46 (4)

### 

Data collection: *APEX2* (Bruker, 2007 ▶); cell refinement: *SAINT* (Bruker, 2007 ▶); data reduction: *SAINT*; program(s) used to solve structure: *SHELXS97* (Sheldrick, 2008 ▶); program(s) used to refine structure: *SHELXL97* (Sheldrick, 2008 ▶); molecular graphics: *XP* in *SHELXTL* (Sheldrick, 2008 ▶) and *DIAMOND* (Brandenburg, 1999 ▶); software used to prepare material for publication: *SHELXTL*.

## Supplementary Material

Crystal structure: contains datablock(s) I, global. DOI: 10.1107/S1600536812042134/hy2592sup1.cif

Structure factors: contains datablock(s) I. DOI: 10.1107/S1600536812042134/hy2592Isup2.hkl

Additional supplementary materials:  crystallographic information; 3D view; checkCIF report

## Figures and Tables

**Table 1:** Hydrogen-bond geometry (Å, °)

*D*—H⋯*A*	*D*—H	H⋯*A*	*D*⋯*A*	*D*—H⋯*A*
N1—H1*A*⋯Cl1^i^	0.90	2.40	3.293 (5)	175
N1—H1*B*⋯Cl2^ii^	0.90	2.71	3.474 (5)	143
N2—H2*A*⋯O3^iii^	0.90	2.52	3.287 (7)	143
N2—H2*B*⋯Cl2^iv^	0.90	2.46	3.310 (5)	157

Symmetry codes: (i) 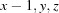; (ii) 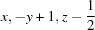; (iii) 
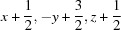
; (iv) 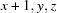.

**Fig. 1.**
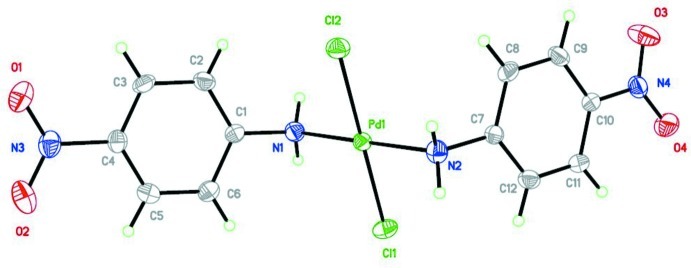
The molecular structure of the title compound, showing 30% probability displacement ellipsoids.

**Fig. 2.**
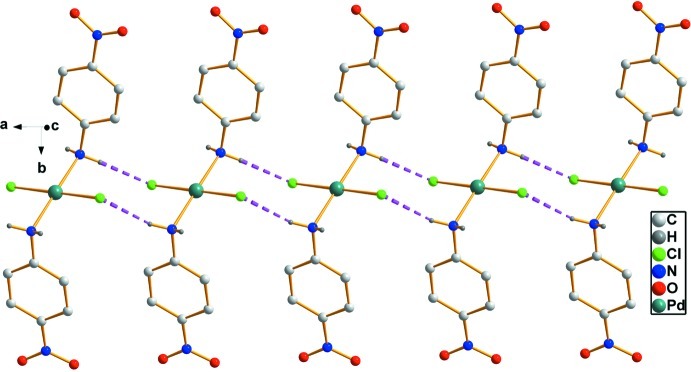
View of the chain structure in the title compound. Hydrogen bonds are shown as pink dashed lines. H atoms not involved in hydrogen bonds have been omitted for clarity.

**Fig. 3.**
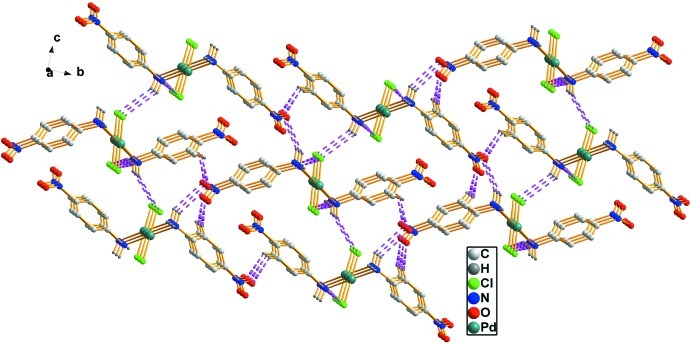
Crystal packing of the title compound. Hydrogen bonds are shown as pink dashed lines. H atoms not involved in hydrogen bonds have been omitted for clarity.

## supplementary crystallographic information

### Comment

Palladium compounds have attracted much attention due to their applications in homogeneous and heterogeneous catalyses (Padmanabhan *et al.*, 1985). Some dramatic results in the homogeneous catalysis of the reactions of organic compounds, particularly the successful commercial exploitation of the Wacker one stage process for the homogeneous catalytic oxidation of ethylene to acetaldehyde in the presence of palladium(II) chloride (Hartley, 1973), have contributed to this interest. In this paper, we report the crystal structure of the title compound, a new palladium(II) complex obtained by the reaction of 4-nitroaniline with palladium chloride in ethanol solution.

As illustrated in Fig. 1, the Pd^II^ atom exhibits a distorted square-planar coordination geometry, defined by two N atoms from two 4-nitroaniline ligands and two chloride atoms. The molecule adopts the *trans* configuration. The bond distances of Pd—N [2.061 (2) Å] and Pd—Cl [2.302 (3) Å] are comparable with the values found in related complexes (Chen *et al.*, 2002; Newkome *et al.*, 1982). The dihedral angle between the aromatic ring plane and the square plane around Pd1 is 70.54 (2)°. Intermolecular N—H···Cl hydrogen bonds involving the amino groups and chlorine anions (Table 1) lead to a chain along [100] (Fig. 2). These chains are further self-assembled into a three-dimensional network through N—H···O and N—H···Cl hydrogen bonds (Fig. 3).

### Experimental

A mixture of palladium chloride (0.1 mmol, 0.018 g) and 4-nitroaniline (0.2 mmol, 0.027 g) in 10 ml of anhydrous ethanol was sealed in an autoclave equipped with a Teflon liner (23 ml) and then heated at 353 K for 2 days. Yellow crystals were obtained by slow evaporation of the solvent at room temperature (yield: 49% based on 4-nitroaniline).

### Refinement

All H atoms were positioned geometrically and refined using a riding model, with C—H = 0.93 and N—H = 0.90 Å and with *U*_iso_(H) = 1.2*U*_eq_(C,N). The hightest peak is located 0.99 Å from Pd1 and the deepest hole is located 1.24 Å from H5.

### Crystal data

**Table:**

[PdCl_2_(C_6_H_6_N_2_O_2_)_2_]	*F*(000) = 896
*M**_r_* = 453.56	*D*_x_ = 1.878 Mg m^−^^3^
Monoclinic, *C**c*	Mo *K*α radiation, λ = 0.71073 Å
Hall symbol: C -2yc	Cell parameters from 5300 reflections
*a* = 5.6014 (8) Å	θ = 1.3–28.0°
*b* = 26.246 (4) Å	µ = 1.51 mm^−^^1^
*c* = 11.0763 (16) Å	*T* = 296 K
β = 99.828 (2)°	Block, yellow
*V* = 1604.5 (4) Å^3^	0.33 × 0.30 × 0.26 mm
*Z* = 4	

### Data collection

**Table:**

Bruker APEXII CCD diffractometer	2135 independent reflections
Radiation source: fine-focus sealed tube	2075 reflections with *I* > 2σ(*I*)
Graphite monochromator	*R*_int_ = 0.018
φ and ω scans	θ_max_ = 25.2°, θ_min_ = 2.4°
Absorption correction: multi-scan (*SADABS*; Sheldrick, 1996)	*h* = −6→6
*T*_min_ = 0.626, *T*_max_ = 0.688	*k* = −28→31
4453 measured reflections	*l* = −13→10

### Refinement

**Table:**

Refinement on *F*^2^	Secondary atom site location: difference Fourier map
Least-squares matrix: full	Hydrogen site location: inferred from neighbouring sites
*R*[*F*^2^ > 2σ(*F*^2^)] = 0.021	H-atom parameters constrained
*wR*(*F*^2^) = 0.077	*w* = 1/[σ^2^(*F*_o_^2^) + (0.059*P*)^2^ + 0.1195*P*] where *P* = (*F*_o_^2^ + 2*F*_c_^2^)/3
*S* = 1.06	(Δ/σ)_max_ = 0.002
2135 reflections	Δρ_max_ = 0.50 e Å^−^^3^
208 parameters	Δρ_min_ = −0.37 e Å^−^^3^
2 restraints	Absolute structure: Flack (1983), 696 Friedel pairs
Primary atom site location: structure-invariant direct methods	Flack parameter: 0.46 (4)

### Special details

**Table:**

Geometry. All e.s.d.'s (except the e.s.d. in the dihedral angle between two l.s. planes) are estimated using the full covariance matrix. The cell e.s.d.'s are taken into account individually in the estimation of e.s.d.'s in distances, angles and torsion angles; correlations between e.s.d.'s in cell parameters are only used when they are defined by crystal symmetry. An approximate (isotropic) treatment of cell e.s.d.'s is used for estimating e.s.d.'s involving l.s. planes.
Refinement. Refinement of *F*^2^ against ALL reflections. The weighted *R*-factor *wR* and goodness of fit *S* are based on *F*^2^, conventional *R*-factors *R* are based on *F*, with *F* set to zero for negative *F*^2^. The threshold expression of *F*^2^ > σ(*F*^2^) is used only for calculating *R*-factors(gt) *etc*. and is not relevant to the choice of reflections for refinement. *R*-factors based on *F*^2^ are statistically about twice as large as those based on *F*, and *R*- factors based on ALL data will be even larger.

### Fractional atomic coordinates and isotropic or equivalent isotropic displacement parameters (Å^2^)

**Table:**

	*x*	*y*	*z*	*U*_iso_*/*U*_eq_	
C1	0.7813 (8)	0.46008 (19)	0.2257 (5)	0.0324 (10)	
C2	0.6105 (10)	0.4507 (2)	0.2984 (6)	0.0405 (13)	
H2	0.4896	0.4745	0.3037	0.049*	
C3	0.6200 (9)	0.4053 (2)	0.3641 (5)	0.0406 (12)	
H3	0.5098	0.3985	0.4161	0.049*	
C4	0.7981 (9)	0.37081 (19)	0.3496 (5)	0.0358 (11)	
C5	0.9644 (10)	0.3795 (2)	0.2764 (5)	0.0403 (12)	
H5	1.0798	0.3547	0.2692	0.048*	
C6	0.9651 (10)	0.4245 (2)	0.2123 (5)	0.0380 (12)	
H6	1.0801	0.4311	0.1629	0.046*	
C7	1.1775 (9)	0.66743 (19)	0.2754 (5)	0.0313 (10)	
C8	0.9834 (10)	0.6949 (2)	0.3044 (5)	0.0371 (11)	
H8	0.8961	0.6828	0.3628	0.045*	
C9	0.9216 (10)	0.7412 (2)	0.2439 (5)	0.0380 (12)	
H9	0.7917	0.7605	0.2606	0.046*	
C10	1.0586 (8)	0.75750 (18)	0.1588 (4)	0.0307 (10)	
C11	1.2582 (9)	0.73067 (19)	0.1320 (5)	0.0358 (11)	
H11	1.3503	0.7432	0.0762	0.043*	
C12	1.3145 (9)	0.6845 (2)	0.1918 (5)	0.0366 (11)	
H12	1.4445	0.6653	0.1753	0.044*	
Cl1	1.2293 (4)	0.55635 (8)	0.0903 (2)	0.0436 (6)	
Cl2	0.7793 (4)	0.56838 (7)	0.4000 (2)	0.0408 (5)	
N1	0.7776 (8)	0.50756 (15)	0.1568 (4)	0.0356 (9)	
H1A	0.6253	0.5198	0.1427	0.043*	
H1B	0.8208	0.5010	0.0837	0.043*	
N2	1.2299 (8)	0.61827 (16)	0.3332 (4)	0.0365 (9)	
H2A	1.2109	0.6202	0.4122	0.044*	
H2B	1.3851	0.6099	0.3316	0.044*	
N3	0.8016 (11)	0.3224 (2)	0.4165 (6)	0.0463 (15)	
N4	0.9877 (9)	0.80407 (15)	0.0883 (5)	0.0404 (10)	
O1	0.6631 (11)	0.3158 (2)	0.4860 (6)	0.0769 (16)	
O2	0.9473 (9)	0.28974 (16)	0.3938 (5)	0.0609 (12)	
O3	0.7966 (11)	0.8248 (2)	0.1012 (5)	0.0674 (18)	
O4	1.1159 (9)	0.82003 (16)	0.0195 (5)	0.0601 (12)	
Pd1	1.00552 (7)	0.562350 (12)	0.24606 (5)	0.02914 (13)	

### Atomic displacement parameters (Å^2^)

**Table:**

	*U*^11^	*U*^22^	*U*^33^	*U*^12^	*U*^13^	*U*^23^
C1	0.027 (2)	0.035 (3)	0.035 (3)	−0.0012 (19)	0.005 (2)	0.000 (2)
C2	0.029 (3)	0.037 (3)	0.056 (4)	0.001 (2)	0.010 (3)	−0.008 (3)
C3	0.034 (3)	0.045 (3)	0.047 (3)	−0.006 (2)	0.020 (2)	−0.003 (3)
C4	0.038 (3)	0.032 (3)	0.036 (3)	−0.006 (2)	0.004 (2)	−0.002 (2)
C5	0.039 (3)	0.041 (3)	0.042 (3)	0.006 (2)	0.010 (2)	−0.003 (2)
C6	0.038 (3)	0.044 (3)	0.033 (3)	−0.003 (2)	0.011 (2)	0.002 (2)
C7	0.029 (2)	0.029 (2)	0.036 (2)	−0.0016 (19)	0.005 (2)	−0.005 (2)
C8	0.040 (3)	0.039 (3)	0.036 (3)	−0.005 (2)	0.016 (2)	0.004 (2)
C9	0.035 (2)	0.033 (3)	0.046 (3)	0.0080 (19)	0.007 (3)	−0.005 (2)
C10	0.028 (2)	0.031 (2)	0.033 (3)	−0.0011 (18)	0.006 (2)	0.000 (2)
C11	0.033 (2)	0.038 (3)	0.039 (3)	0.000 (2)	0.013 (2)	−0.001 (2)
C12	0.029 (2)	0.040 (3)	0.041 (3)	0.004 (2)	0.007 (2)	−0.002 (2)
Cl1	0.0384 (10)	0.0581 (11)	0.0374 (11)	0.0009 (7)	0.0154 (8)	0.0016 (7)
Cl2	0.0420 (10)	0.0447 (9)	0.0380 (11)	0.0022 (6)	0.0133 (8)	−0.0019 (7)
N1	0.033 (2)	0.035 (2)	0.038 (2)	−0.0002 (17)	0.0059 (18)	−0.0011 (18)
N2	0.036 (2)	0.036 (2)	0.036 (2)	0.0006 (17)	0.0009 (18)	0.0037 (18)
N3	0.059 (3)	0.043 (3)	0.037 (3)	−0.003 (3)	0.008 (3)	0.004 (2)
N4	0.045 (2)	0.031 (2)	0.045 (3)	0.0020 (19)	0.007 (2)	0.001 (2)
O1	0.084 (4)	0.069 (3)	0.087 (4)	−0.002 (3)	0.040 (3)	0.028 (3)
O2	0.076 (3)	0.044 (2)	0.056 (3)	0.008 (2)	−0.005 (2)	0.006 (2)
O3	0.086 (4)	0.068 (4)	0.057 (3)	0.042 (3)	0.036 (3)	0.024 (3)
O4	0.064 (3)	0.053 (3)	0.071 (3)	0.004 (2)	0.031 (2)	0.023 (2)
Pd1	0.02850 (17)	0.02877 (19)	0.03039 (19)	0.00214 (16)	0.00572 (12)	0.00207 (17)

### Geometric parameters (Å, º)

**Table:**

C1—C2	1.374 (8)	C9—H9	0.9300
C1—C6	1.416 (8)	C10—C11	1.395 (7)
C1—N1	1.459 (7)	C10—N4	1.468 (6)
C2—C3	1.394 (9)	C11—C12	1.390 (7)
C2—H2	0.9300	C11—H11	0.9300
C3—C4	1.376 (8)	C12—H12	0.9300
C3—H3	0.9300	Cl1—Pd1	2.305 (2)
C4—C5	1.354 (8)	Cl2—Pd1	2.297 (2)
C4—N3	1.470 (8)	N1—Pd1	2.060 (4)
C5—C6	1.379 (8)	N1—H1A	0.9000
C5—H5	0.9300	N1—H1B	0.9000
C6—H6	0.9300	N2—Pd1	2.062 (4)
C7—C12	1.375 (8)	N2—H2A	0.9000
C7—C8	1.387 (7)	N2—H2B	0.9000
C7—N2	1.448 (7)	N3—O1	1.196 (9)
C8—C9	1.403 (8)	N3—O2	1.238 (8)
C8—H8	0.9300	N4—O4	1.207 (7)
C9—C10	1.381 (8)	N4—O3	1.231 (7)
			
C2—C1—C6	122.3 (5)	C12—C11—C10	117.6 (5)
C2—C1—N1	120.6 (5)	C12—C11—H11	121.2
C6—C1—N1	117.0 (5)	C10—C11—H11	121.2
C1—C2—C3	119.4 (5)	C7—C12—C11	119.9 (5)
C1—C2—H2	120.3	C7—C12—H12	120.1
C3—C2—H2	120.3	C11—C12—H12	120.1
C4—C3—C2	117.7 (5)	C1—N1—Pd1	113.1 (3)
C4—C3—H3	121.1	C1—N1—H1A	109.0
C2—C3—H3	121.1	Pd1—N1—H1A	109.0
C5—C4—C3	123.1 (5)	C1—N1—H1B	109.0
C5—C4—N3	119.7 (5)	Pd1—N1—H1B	109.0
C3—C4—N3	117.2 (5)	H1A—N1—H1B	107.8
C4—C5—C6	120.9 (5)	C7—N2—Pd1	111.5 (3)
C4—C5—H5	119.5	C7—N2—H2A	109.3
C6—C5—H5	119.5	Pd1—N2—H2A	109.3
C5—C6—C1	116.5 (5)	C7—N2—H2B	109.3
C5—C6—H6	121.8	Pd1—N2—H2B	109.3
C1—C6—H6	121.8	H2A—N2—H2B	108.0
C12—C7—C8	122.4 (5)	O1—N3—O2	123.6 (6)
C12—C7—N2	119.7 (5)	O1—N3—C4	119.7 (6)
C8—C7—N2	117.9 (5)	O2—N3—C4	116.6 (6)
C7—C8—C9	118.6 (5)	O4—N4—O3	122.9 (5)
C7—C8—H8	120.7	O4—N4—C10	119.2 (5)
C9—C8—H8	120.7	O3—N4—C10	117.9 (5)
C10—C9—C8	118.3 (5)	N1—Pd1—N2	178.86 (17)
C10—C9—H9	120.8	N1—Pd1—Cl2	91.68 (14)
C8—C9—H9	120.8	N2—Pd1—Cl2	88.43 (15)
C9—C10—C11	123.2 (5)	N1—Pd1—Cl1	87.97 (15)
C9—C10—N4	119.3 (4)	N2—Pd1—Cl1	91.91 (15)
C11—C10—N4	117.5 (4)	Cl2—Pd1—Cl1	179.48 (11)

### Hydrogen-bond geometry (Å, º)

**Table:**

*D*—H···*A*	*D*—H	H···*A*	*D*···*A*	*D*—H···*A*
N1—H1*A*···Cl1^i^	0.90	2.40	3.293 (5)	175
N1—H1*B*···Cl2^ii^	0.90	2.71	3.474 (5)	143
N2—H2*A*···O3^iii^	0.90	2.52	3.287 (7)	143
N2—H2*B*···Cl2^iv^	0.90	2.46	3.310 (5)	157

Symmetry codes: (i) *x*−1, *y*, *z*; (ii) *x*, −*y*+1, *z*−1/2; (iii) *x*+1/2, −*y*+3/2, *z*+1/2; (iv) *x*+1, *y*, *z*.

## References

[bb1] Brandenburg, K. (1999). *DIAMOND* Crystal Impact GbR, Bonn, Germany.

[bb2] Bruker (2007). *APEX2* and *SAINT* Bruker AXS Inc., Madison, Wisconsin, USA.

[bb3] Chen, Y.-B., Li, Z.-J., Qin, Y.-Y., Kang, Y., Wu, L. & Yao, Y.-G. (2002). *Chin. J. Struct. Chem.* **21**, 530–532.

[bb4] Flack, H. D. (1983). *Acta Cryst.* A**39**, 876–881.

[bb5] Hartley, F. R. (1973). *The Chemistry of Platinum and Palladium* New York: John Wiley and Sons.

[bb6] Newkome, G. R., Fronczek, F. R., Grupta, V. K., Puckett, W. E., Pantaleo, D. C. & Kiefer, G. E. (1982). *J. Am. Chem. Soc.* **104**, 1782–1783.

[bb7] Padmanabhan, V. M., Patel, R. P. & Ranganathan, T. N. (1985). *Acta Cryst.* C**41**, 1305–1307.

[bb8] Sheldrick, G. M. (1996). *SADABS* University of Göttingen, Germany.

[bb9] Sheldrick, G. M. (2008). *Acta Cryst.* A**64**, 112–122. 10.1107/S0108767307043930 18156677

